# The relationship between tumor-infiltrating neutrophils and clinical outcomes in patients with resectable hepatocellular carcinoma

**DOI:** 10.1186/s12885-024-12074-3

**Published:** 2024-03-11

**Authors:** Jung Hee Lee, Young Mi Hong

**Affiliations:** 1 Department of Pathology, Pusan National University School of Medicine, Pusan National University Yangsan Hospital, Yangsan, Republic of Korea; 2 Department of Internal Medicine, Pusan National University School of Medicine, Pusan National University Yangsan Hospital, Yangsan, Republic of Korea

**Keywords:** HCC, Neutrophils, Tumor microenvironment, Prognosis, Resection

## Abstract

**Background:**

The impact of tumor-infiltrating neutrophils (TINs) on clinical outcomes has been reported in various cancer types, but their role in hepatocellular carcinoma (HCC) has not been fully evaluated. The aim of this study was to investigate the prognostic values for TINs in HCC patients undergoing curative resection.

**Methods:**

We assessed immune markers (CD3, CD4, CD8, CD66b) using immunohistochemistry in 115 patients who underwent curative resection for HCC. We analyzed the prognostic values for tumor-infiltrating immune cells, including neutrophils, and other clinicopathological factors.

**Results:**

In the Multivariate Cox analysis of overall survival (OS), alpha-fetoprotein (AFP) ≥ 100 ng/mL (hazard ratio (HR), 2.74, 95% confidence interval (CI), 1.17–6.44; *P* = 0.021) and Barcelona Clinic Liver Cancer (BCLC) B/C stage (HR, 3.98, 95% CI, 1.68–9.43; *P* = 0.020) were found to be independent poor prognostic factors in HCC patients undergoing resection. The presence of CD66b^+^TINs was observed in 66 (57.4%) patients. However, CD66b^+^TINs were not associated with recurrence-free survival and OS.

**Conclusions:**

Our study identified low CD66b^+^TINs in resectable HCC, and CD66b^+^ TINs did not have a significant role for the clinical outcomes of patients undergoing curative resection. The results suggest that TINs may play a role in more advanced stages of HCC.

## Background

Hepatocellular carcinoma (HCC) is one of the most common cancer and third leading cause of cancer death worldwide [1]. Although hepatic resection is the treatment of choice as a curative purpose in HCC*,* high recurrence rate limit the curative purpose in most patients [2]. Predicting the prognosis, including recurrence, is crucial in identifying appropriate interventions that can improve patients outcomes. Various clinical and pathological factors have been reported to predict the prognosis, such as tumor marker, tumor stage, and tumor differentiation grade. In recent years, numerous inflammatory markers have also been suggested as useful for predicting prognosis in various cancers [3–5].

There is growing evidence suggesting a link between inflammation and cancer [6, 7]. Tumor-infiltrating immune cells, including T cells, B cells, natural killer cells, and myeloid lineage leukocytes, are considered essential components of the host immune response against cancer, contributing to either pro-tumoral or anti-tumoral immunity. Several studies have reported the potential role of tumor-infiltrating immune cells as a prognostic factor for various malignancies [8–12].

Neutrophils are short-lived effector cells that play a crucial role in the immune system`s response to infections and inflammation. However, due to their short lifespan and non-proliferative characteristics, their essential role in cancer has often been underestimated. In the context of cancer, neutrophils can have both pro-tumor and anti-tumor effects, depending on the stage and type of cancer [13, 14]. They can promote tumor growth by releasing growth factors and inflammatory molecules that stimulate angiogenesis and promote tumor cell survival. On the other hand, they can also suppress the immune system`s response to the tumor, which can help the tumor evade detection and elimination by the immune system.

Previous research has shown that neutrophil to lymphocyte ratio (NLR) can be a useful biomarker for predicting cancer prognosis and response to treatment. Many studies have reported that a high NLR has been associated with poor outcomes in HCC [15]. Additionally, some studies have suggested the role of tumor-infiltrating neutrophils (TINs) in the local microenvironment [8–10, 12, 16, 17]. However, the prognostic role of TINs remains controversial due to the plasticity of neutrophil in the tumor microenvironment (TME). Furthermore, there has been no report regarding the correlation between the peripheral neutrophils and TINs. Therefore, in this study, we aimed to investigate the clinical significance of TINs and the correlation between the peripheral neutrophil and TINs in HCC undergone curative resection.

## Patients and methods

### Patients

We conducted a retrospective analysis of patients who underwent curative resection between 2009 and 2012. Curative resection was defined as removal of the tumor with a tumor-free margin and no residual tumors in the remaining liver. Patients were followed up after the hepatic resection, and laboratory measurements including alpha-fetoprotein (AFP) and dynamic computed tomography (CT) or magnetic resonance imaging were performed every 3 months for first 2–3 years and then every 6 months. Treatment for tumor recurrence followed generally accepted guidelines [18, 19]. The median follow-up duration was 101.5 months (range, 1–138 months). Recurrence free survival (RFS) was defined as the length of time from hepatic resection to recurrence or death. Overall survival (OS) was defined as the interval between the dates of hepatic resection and death.

### Clinicopathological analysis

We reviewed the demographics and laboratory test findings of patients, including age, gender, hepatitis status, tumor marker (AFP), and NLR. The NLR was defined as the absolute neutrophil count divided by the absolute lymphocyte count. We also reviewed the histopathological findings of the HCC, such as tumor size, tumor number, tumor differentiation, vascular invasion, and non-tumor liver pathology. The histologic tumor differentiation was determined according to the criteria of Edmondson and Steiner [20]. Grades I and II were considered well and moderately differentiated HCC, respectively, and grades III and IV were considered as poor-differentiated HCC.

### Immunohistochemical study and quantitative analysis

Representative 4-µm sections of formalin-fixed paraffin-embedded tissues were cut on charged glass slides. Slides were placed in a 65 $^\circ{\rm C}$ oven for 20 min to dry tissues. Immunostaining was performed using a Bond-Max (Leica, Wetzlar, Germany) or a Ventana Benchmark ULTRA (Roche Diagnostics, Basel, Switzerland) staining instrument as described by the manufacturer. Briefly, paraffin wax was removed using Bond Dewax Solution (AR9222, Novocastra, Newcastle upon Tyne, UK) or EZ prep solution (Ventana Medical Systems, Mountain View, CA, USA) at 72 $^\circ{\rm C}$ and sections were rehydrated. Heat-induced epitope retrieval was achieved using Bond Epitope Retrieval Solution 2 (EDTA-based buffer) for 20 min at 100 $\mathrm{^\circ{\rm C} }$ or Ultra Cell Conditioning Solution 1 (Ventana Medical Systems, Mountain View, CA, USA) for 64 min at 100 $\mathrm{^\circ{\rm C} }$. Sections were then incubated with antibodies CD3 (A0452 polyclonal, 1:200; DAKO, Denmark) and CD66b (ab197678 polyclonal, 1:200; Abcam) for 20 min at room temperature. Monoclonal antibodies CD4 (790–4423 clone No. SP35; Roche Ventana) and CD8 (790–4460 clone No. SP57; Roche Ventana) which are prediluted ready-to-use solutions for Ventana Benchmark automated staining system were diluted 1:2 and used for Bond-Max system. Finally, 3,3′-diaminobenzidine tetrahydrochloride was used as the chromogen. The human tonsil tissue was used as a positive control.

Whole-slide images (WSIs) of CD3, CD4, CD8, and CD66b staining were acquired using the Pannoramic 250 Flash III (3DHistech Ltd., Budapest, Hungary), analyzed using the 3DHistech CaseViewer software and antibody-positive cells were counted using the 3DHistech QuantCenter module.

### Statistical analysis

RFS and OS were calculated using Kaplan–Meier method, and statistical evaluation was performed using a log-rank test. Multivariate Cox proportional hazards regression model was used to evaluate the association between clinicopathological factors and survival. All statistical analyses were performed with SPSS software 21.0 (SPSS Inc., Chicago, IL, USA). *P*-values of less than 0.05 were considered statistically significant.

## Results

### Patient characteristics

This study included 115 patients with HCC who underwent curative hepatectomy. The clinicopathological factors are summarized in Table 1. The median age was 59 years (range, 31–80), and the majority of patients were male (80%). Among the patients, 80% had hepatitis B virus, and 63.5% of HCC patients had underlying liver cirrhosis. 76 patients (66.1%) had early-stage HCC (Barcelona Clinic Liver Cancer (BCLC) 0/A stage). 43.5% presented with well/moderate differentiated HCC, and vascular invasion was identified in 27.8% of patients.

**Table 1:** Patients characteristics

Variables	Total = 115
Age (years), n (%)	
< 60	64 (55.7)
≥ 60	51 (44.3)
Gender, n (%)	
Female	23 (20.0)
Male	92 (80.0)
Etiology, n (%)	
HBV	92 (80.0)
Others	23 (20.0)
AFP (ng/mL), n (%)	
< 100	64 (55.7)
≥ 100	51 (44.3)
Tumor size (cm), n (%)	
≤ 3	57 (49.6)
< 3 ~ ≤ 5	24 (20.9)
> 5	34 (29.6)
Tumor number, n (%)	
Single	106 (92.2)
Multiple	9 (7.8)
TMN stage, n (%)	
I	18 (15.7)
II	52 (45.2)
III	45 (39.1)
BCLC stage, n (%)	
0/A	76 (66.1)
B/C	39 (33.9)
ES grade, n (%)	
Well/Moderate	50 (43.5)
Poor	65 (56.5)
Vascular invasion, n (%)	
Absent	83 (72.2)
Present	32 (27.8)
Underlying cirrhosis, n (%)	
Absent	42 (36.5)
Present	73 (63.5)

*AFP* Alpha-fetoprotein, *BCLC* Barcelona Clinic Liver Cancer, *ES* Edmondson and Steiner

### Association between clinicopathological factors and prognosis

The median duration of follow-up was 101.5 months (range, 1–138 months). The 1-year, 3-year, 5-year and 8 year OS were 89.6%, 72.9%, 72.0%, and 61.5% respectively. The 1-year, 3-year, 5-year and 8 year RFS were 92.1%, 71.5%, 55.2%, and 43.7% respectively.

To identify clinically significant factors, univariate analysis was conducted on survival data. In the univariate analysis, AFP (*P* = 0.016) and BCLC stage (*P* = 0.001) were associated with OS. Subsequently, both AFP and BCLC stage, which showed significance in the univariate analysis, were included in the multivariate Cox proportional hazards regression model. In the multivariate analysis, both AFP ≥ 100 ng/mL (HR, 2.74, 95% CI, 1.17–6.44; *P* = 0.021) and BCLC B/C stage (HR, 3.98, 95% CI, 1.68–9.43; *P* = 0.020) were associated with poor OS (Table 2). However, no prognostic factors were found to be associated with RFS (Table 3).

**Table 2:** Univariate and multivariate Cox regression analyses of overall survival

Variables	HR(95% CI)	*P* value
**Univariate**		
Age:≥ 60 yrs vs< 60 yrs	1.94(0.87–4.30)	0.105
Gender: male vs female	1.37(0.49–3.83)	0.547
Etiology: HBV vs others	2.30(0.84–6.28)	0.104
AFP:≥ 100 ng/mL vs < 100 ng/mL	2.71(1.20–6.10)	0.016
BCLC stage: B/C vs 0/A	3.95(1.71–9.10)	0.001
ES grade: poor vs well/moderate	1.76(0.78–4.01)	0.177
Underlying cirrhosis: yes vs no	2.15(0.90–5.17)	0.087
CD 66b expression: present vs absent	0.61(0.28–1.36)	0.227
NLR:≥ 2.5 vs< 2.5	1.67(0.75–3.71)	0.206
**Multivariate**		
Age:≥ 60 vs< 60		
Gender: male vs female		
Etiology: HBV vs others		
AFP:≥ 100 ng/mL vs< 100 ng/mL	2.74(1.17–6.44)	0.021
BCLC stage: B/C vs 0/A	3.98(1.68–9.43)	0.020
ES grade: poor vs well/moderate		
Underlying cirrhosis: no vs yes		
CD 66b expression: present vs absent		
NLR:≥ 2.5 vs< 2.5		

*AFP* Alpha-fetoprotein, *BCLC* Barcelona Clinic Liver Cancer, *CI* Confidence interval, *ES* Edmondson and Steiner, *HR* Hazard ratio, *NLR* Neutrophil to lymphocyte ratio

**Table 3:** Univariate Cox regression analyses of recurrence free survival

Variables	HR(95% CI)	*P* value
**Univariate**		
Age:≥ 60 yrs vs< 60 yrs	1.62(0.64–4.06)	0.307
Gender: male vs female	1.62(0.64–4.06)	0.307
Etiology: HBV vs others	0.79(0.29–2.14)	0.643
AFP:≥ 100 ng/mL vs< 100 ng/mL	0.99(0.48–2.08)	0.984
BCLC stage: B/C vs 0/A	2.78(1.23–6.29)	0.014
ES grade: poor vs well/moderate	0.75(0.36–1.58)	0.448
Underlying cirrhosis: yes vs no	1.51(0.70–3.26)	0.292
CD 66b expression: present vs absent	1.25(0.59–2.63)	0.560
NLR:≥ 2.5 vs< 2.5	0.97(0.47–2.01)	0.930

*AFP* Alpha-fetoprotein, *BCLC* Barcelona Clinic Liver Cancer, *CI* Confidence interval, *ES* Edmondson and Steiner, *HR* Hazard ratio, *NLR* Neutrophil to lymphocyte ratio

Although we investigated the relationship between the expression of CD3, CD4, CD8 and CD66b in HCC and the clinicopathological features to further characterize the function of tumor infiltrating immune cells in HCC patients, we did not find any correlation (Table 4).

**Table 4:** Correlations of CD3/CD4/CD8/CD66b density with clinicopathological characteristics

	**CD3**			**CD4**			**CD8**			**CD66b**		
**Variables**	**Low (*n* = 58)**	**High (*n* = 57)**	***P* value**	**Low (*n* = 57)**	**High (*n* = 58)**	***P* Value**	**Low (*n* = 57)**	**High (*n* = 58)**	***P* value**	**Low (*n* = 46)**	**High (*n* = 69)**	***P* value**
**Age**			0.218			0.786			0.917			0.540
< 60 yrs	29(50.0%)	35(61.4%)		31(54.4%)	33(56.9%)		32(56.1%)	32(55.2%)		24(52.2%)	40(58.0%)	
≥ 60 yrs	29(50.0%)	22(38.6%)		26(45.6%)	25(43.1%)		25(43.9%)	26(44.8%)		22(47.8%)	29(42.0%)	
**Gender**			0.225			0.263			0.113			0.046
Male	49(84.5%)	43(75.4%)		48(84.2%)	44(75.9%)		49(86.0%)	43(74.1%)		41(89.1%)	51(73.9%)	
Female	9(15.5%)	14(24.6%)		9(15.8%)	14(24.1%)		8(14.0%)	15(25.9%)		5(10.9%)	18(26.1%)	
**Etiology**			0.225			0.225			0.834			0.081
HBV	46(79.3%)	50(87.7%)		50(87.7%)	46(79.3%)		48(84.2%)	48(82.8%)		35(76.1%)	61(88.4%)	
Others	12(20.7%)	7(12.3%)		7(12.3%)	12(20.7%)		9(15.8%)	10(17.2%)		11(23.9%)	8(11.6%)	
**AFP**			0.162			0.108			0.392			0.358
< 100 ng/mL	36(62.1%)	28(49.1%)		36(63.2%)	28(48.3%)		34(59.6%)	30(51.7%)		28(60.9%)	36(52.2%)	
≥ 100 ng/mL	22(25.7%)	29(50.9%)		21(36.8%)	30(51.7%)		23(25.3%)	28(48.3%)		18(39.1%)	33(47.8%)	
**Tumor size**			0.115			0.639			0.198			0.505
≤ 5 cm	37(40.9%)	44(40.1%)		39(68.4%)	42(72.4%)		37(64.9%)	44(75.9%)		34(73.9%)	47(68.1%)	
> 5 cm	21(36.2%)	13(22.8%)		18(31.6%)	16(27.6%)		20(35.1%)	14(24.1%)		12(26.1%)	22(31.9%)	
**Tumor number**			0.749			0.749			0.708			0.777
Single	53(91.4%)	53(93.0%)		53(93.0%)	53(91.4%)		52(91.2%)	54(93.1%)		42(91.3%)	64(92.8%)	
Multiple	5(8.6%)	4(7.0%)		4(7.0%)	5(8.6%)		5(8.8%)	4(6.9%)		4(8.7%)	5(7.2%)	
**ES grade**			0.518			0.306			0.914			0.817
Well/Moderate	23(39.7%)	26(45.6%)		27(47.4%)	22(37.9%)		24(42.1%)	25(43.1%)		19(41.3%)	30(43.5%)	
Poor	35(60.3%)	31(54.4%)		30(52.6%)	36(62.1%)		33(57.9%)	33(56.9%)		27(58.7%)	39(56.5%)	
**Vascular invasion**			0.954			0.635			0.191			0.445
Absent	42(72.4%)	41(71.9%)		40(70.2%)	43(74.1%)		38(66.7%)	45(77.6%)		35(76.1%)	48(69.6%)	
Present	16(27.6%)	16(28.1%)		17(29.8%)	15(25.9%)		19(33.3%)	13(22.4%)		11(23.9%)	21(30.4%)	

*AFP* Alpha-fetoprotein, *ES* Edmondson and Steiner

### Relationship between TINs expressing CD66b and survival

**Fig. 1:**
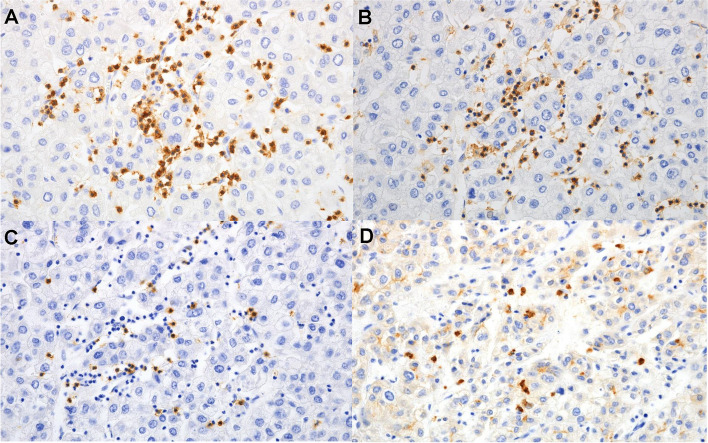
Representative images of tumor-infiltrating immune cells in the tumor tissues ( **A**,CD3;**B**, CD4;**C**, CD8;**D**,CD66b staining)

**Fig. 2:**
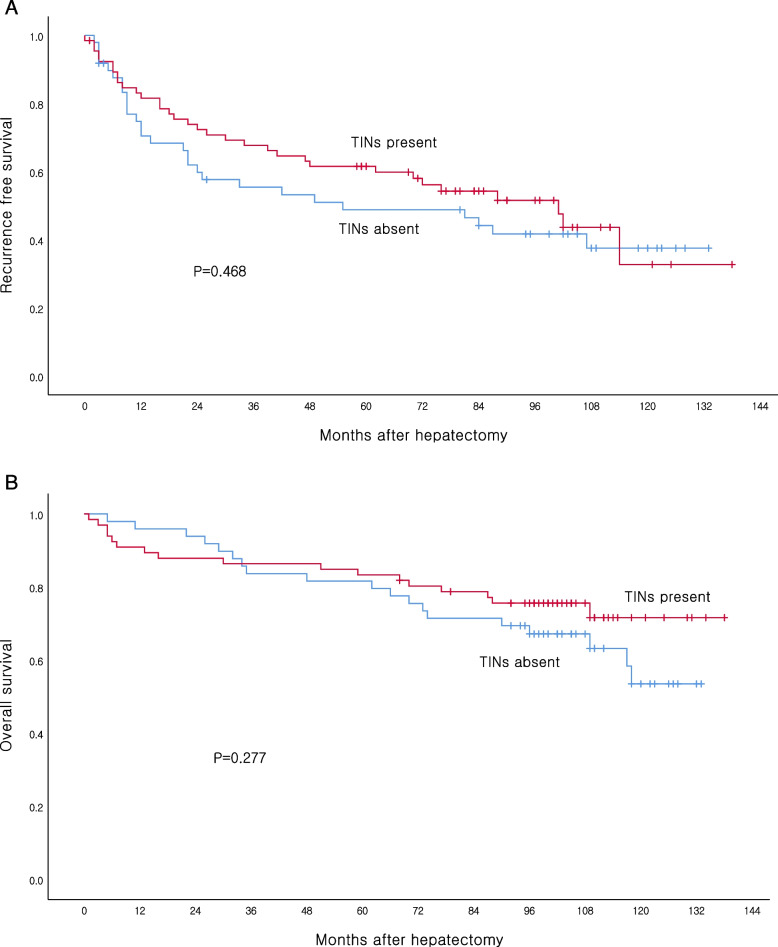
**A** Kaplan–Meier analysis of recurrence free survival and **B** overall survival in in HCC according to TINs

The densities of CD3, CD4, CD8 and CD66b-expressing immune cells were analyzed in the tumor tissues (Fig. 1). While CD66b^+^TINs was present in 66 (57.4%) patients, there were no statistically significant differences in RFS and OS according to TINs (Fig. 2). Survival analysis was also performed based on CD3, CD4 and CD8 expression; however, no significant association with survival were observed (data not shown). Additionally, there was no correlation found between the peripheral neutrophil level and TINs in patients with resectable HCC.

## Discussion

Neutrophils play active roles in the TME and exhibit various prognostic effects depending on the cancer types and treatment methods [13]. In this study, we investigated the long-term prognostic value of TINs in patients undergoing curative resection for HCC. Our findings indicate that TINs were not associated with survival.

The correlation between inflammation and cancer has been widely accepted [7], as inflammation predisposes to cancer development and promotes all stages of cancer progression. Cancers develop within a complex and dynamic TME that influences tumor growth, invasion, and metastasis. The TME consists of cancer cells, as well as surrounding stromal and immune cells, which are significant components of the TME. Immune cells are highly plastic due to the reciprocal interactions within the TME, continuously changing their phenotypic and functional characteristics into either pro-tumoral or anti-tumoral immunity [21]. To date, many studies have unveiled the prognostic significance of tumor infiltrating immune cells*,* such as B cells, CD8^+^ tumor infiltrating lymphocyte, T-regulatory cells, tumor associated macrophages, natural killer cells, and myeloid derived suppressor cells [22].

Neutrophils play a role in various diseases, including infectious diseases, metabolic, and autoimmune diseases. Neutrophils, as the most dominant immune cells, also play complicated and significant roles in cancer. In various inflammatory models, they have been shown to be essential for CD8 + T cells recruitment, priming, and activation [23]. Neutrophils exert both anti-tumoral and pro-tumoral functions in the initiation, growth and metastasis of cancer, and these functions are influence by different neutrophil phenotypes. In response to cytokine stimulation, neutrophils develop the ability to polarize to an anti-tumor (N1) or pro-tumor (N2) phenotype [24, 25]. The functional plasticity of neutrophils is regulated by molecules in the TME [13, 21].

Clinical data suggests that neutrophils contribute to the tumor development, and numerous studies have reported the prognostic significance of TINs in various cancers [8–10, 26–29]. The pathophysiological mechanism of HCC is not completely understood, but it is thought to result from a multistep process involving molecular alteration, genomic instability, and chronic inflammation [30]. Tumor-related chronic inflammation alters the TME by promoting the infiltration of several immune cell populations, which promotes HCC development [7]. The immunological characteristics of HCC are complex and vary dynamically due to underlying chronic inflammation and cirrhosis in most HCC patients [31].

Only three studies have investigated the prognostic value of tumor-associated neutrophils in HCC, and the results are not entirely consistent. Li et al. showed that the presence of CD66b^+^ intratumoral neutrophils was a poor prognostic factor for HCC after resection [32], and Wang et al. reported that CD66b^+^intratumoral neutrophils infiltration was an independent prognostic factor for poor survival for HCC, which is consistent with the study by Li et al. [16]. Schoenberg et al. investigated the prognostic value of tumor infiltrating leukocytes in the perivascular region and found that CD66^+^ cells were not significant, while perivascular-infiltrating CD3^+^, CD8^+^, and CD20^+^ cells independently predicted OS and disease free survival [17]. However, our results did not find any prognostic value of CD66^+^ TINs. There could be several possible explanations for this. One possible explanation is that our sample size may not have been large enough to detect a statistically significant difference. Another possible explanation is that the patient population included in our study had less advanced HCC compared to previous studies, and TINs may be more abundant in advanced cancer compared to early-stage cancer [9, 28, 32]. In addition, TINs may interact with other factors, such as tumor infiltrating lymphocytes, cytokines, and chemokines, which could have masked any potential prognostic value of TINs. Overall, the heterogeneity of HCC and its complex immunological characteristics make it difficult to draw definitive conclusions about the prognostic value of TINs, and further research is needed to better understand their role in HCC development and progression.

This study has some limitations, including its retrospective design and small sample size, which may limit the generalizability of the findings. Further prospective studies with larger sample size are needed to confirm these results and explore the functional significance of TINs in HCC. In addition, the study only investigated the association between the level of TINs and clinical outcomes, and further mechanistic studies are needed to better understand the underlying mechanisms involved in the interactions between TINs and HCC.

In conclusion, this study suggests that TINs may not be a significant prognostic factor in HCC, highlighting the complex role of neutrophils in HCC. Further research is needed to gain a more complete understanding of the relationship between neutrophils and HCC, and to develop targeted immunotherapies that can effectively harness the power of the immune system to overcome HCC.

## Data Availability

The datasets generated and/or analysed during the current study are not publicly available due to protect the privacy of the patient, but are available from the corresponding author on reasonable request.

## Abbreviations

AFP: Alpha-fetoprotein
BCLC: Barcelona Clinic Liver Cancer
CI: Confidence interval
CT: Computed tomography
HCC: Hepatocellular carcinoma
HR: Hazard ratio
NLR: Neutrophil to lymphocyte ratio
OS: Overall survival
RFS: Recurrence free survival
TINs: Tumor-infiltrating neutrophils
TME: Tumor microenvironment
